# Mandibular Angle Fractures: Comparison of One Miniplate *vs.* Two Miniplates

**DOI:** 10.5812/traumamon.9865

**Published:** 2013-05-26

**Authors:** Javad Yazdani, Kourosh Taheri Talesh, Mohammad Hosein Kalantar Motamedi, Reza Khorshidi, Sasan Fekri, Saeed Hajmohammadi

**Affiliations:** 1 Department of Oral and Maxillofacial Surgery, Tabriz University of Medical Sciences, Tabriz, IR Iran; 2 Department of Oral and Maxillofacial Surgery, Azad University of Medical Sciences, Tehran, IR Iran; 3 Trauma Research Center, Baqiyatallah University of Medical Sciences, Tehran, IR Iran; 4 Department of Oral and Maxillofacial Surgery, Yazd University of Medical Sciences,Yazd, IR Iran

**Keywords:** Mandibular Fractures, Osteosynthesis, Complications

## Abstract

**Background:**

Monocortical miniplate fixation is an accepted and reliable method for internal fixation of mandibular angle fractures. Although placement of a second miniplate may theoretically provide more stability; however, the clinical importance of this issue remains controversial.

**Objectives:**

The present study assessed the postoperative complications and outcomes associated with the fixation of mandibular angle fractures using 1 and 2 miniplates in patients with favorable mandibular angle fractures.

**Patients and Methods:**

A prospective study of 87 patients (73 males, 14 females) with favorable mandibular angle fractures was done. In the first group, a 4-hole miniplate was placed at the superior border through an intraoral approach. In group 2, patients were treated with 2 miniplates, one placed at the superior border (similar to group 1) and the other on the lateral aspect of the angle at the inferior border through an intraoral and transcutaneous approach using a trocar. Postoperative complications including malocclusion, malunion and sensory disturbances associated with surgery, additional maxillomandibular fixation (MMF) by means of an arch bar and wires for a longer period (for delayed union) and infection were assessed in patients of both groups up to 12 months postoperatively. The data were analyzed using the chi-square test.

**Results:**

In the single miniplate group, 25 patients showed lip numbness associated with surgery (55.6%), 22 patients required additional use of MMF (48.9%) and 3 patients developed infections (6.7%). In the double miniplate group 20 patients showed lip numbness associated with surgery (47.6%), 18 patients required additional use of MMF (42.9%) and 1 patient developed infection (2.4%). None of the patients in either group showed malocclusion or malunion. No significant difference was observed between the groups regarding overall complication rate.

**Conclusions:**

In this study, use of one miniplate or two miniplates for treatment of favorable mandibular angle fractures was associated with a similar incidence of complications. Thus, it seems that the use of two miniplates in this setting may not be warranted, nor cost-efficient.

## 1. Background

Mandibular fractures are not uncommon and have increased significantly in the last decade (1, 2). Mandibular angle fractures account for 23% to 42% of all facial fractures and have a high complication rate (0%-32%) (3, 4). The thin cross-sectional bone area, the presence of the third molars and proximity of tooth roots may cause problems for attaining a stable fixation of the segments. The angle also has limited intraoral access making treatment difficult (5). The applied masticatory forces on the mandibular angle also lead to rotation of the proximal and distal fracture segments and cause displacement of the ramus in unfavorable fractures (6). Most mandibular fractures occur as a result of assault and interpersonal violence and vary among populations; they are also related to increased consumption of alcohol, drug abuse and inadequate oral health (7). Different treatment modalities as well as surgical experience with a specific treatment procedure are important factors influencing the incidence of complications (6). Different treatment modalities have been proposed for mandibular angle fractures; although the ideal modality remains controversial (8-10). Two main procedures are basically used. Internal fixation using a miniplate placed on the external oblique ridge intraorally with or without another miniplate through an intraoral and extraoral approach (11). The advantages of the rigid intraoral fixation as compared with closed reduction techniques are: shorter MMF period or no MMF, early mandibular function, increased patient satisfaction, decreased hospital stay and faster healing (12).

## 2. Objectives

The objective of the present study was to compare the postoperative complication rates after fixation of undisplaced mandibular angle fractures with 1 versus 2 miniplates.

## 3. Patients and Methods

In this prospective study, 87 patients (73 males, 14 females) with isolated mandibular angle fractures were treated and assessed. In the first group, a 4-hole miniplate was placed at the superior border through an intraoral approach. In group 2 the patients were treated with 2 miniplates, one placed at the superior border (similar to group 1) and the other on the lateral aspect of the angle at the inferior border through an intraoral and transcutaneous approach using a trocar. Patients were aged 16-60 years with sufficient dentition to reproduce the occlusion. The study was approved by our local institutional review board and ethics committee because it was retrospective. General anesthesia was administered via nasotracheal intubation. The occlusion was re-established and the maxillomandibular fixation was achieved through application of the arch bars. Third molars in the fracture site were not removed in our patients.The incision line was made on the external oblique ridge. Dissection was continued towards bone using a Freer to identify the fracture site. In both groups, four-hole non-compression miniplates were placed through the intraoral approach on the superior border of the external oblique ridge using monocortical screws.In group 2, a 5-mm incision was made on the skin of the mandibular angle by means of a scalpel. Blunt dissection was continued to the buccal tissues using a Kelly forceps, and then a transbuccal trocar was placed through the skin adjacent to the fracture site. The holes were drilled perpendicular to the fracture using the trocar and the second 4-hole miniplate was secured to the lateral aspect of the mandibular angle across the fracture line using monocortical screws by the transcutaneous approach. The wounds were closed with polygalactin 3-0 sutures and copious irrigation. Then, MMF was released and the arch bars were left in place for heavy gauge elastics. We did not use drains in our patients. We applied MMF to all of our patients for 2 weeks. Antibiotic therapy included 1g Cefazolin intravenously 1 hour preoperatively continued for 2 days with 1g Cefazolin intravenously for every 8 hours and Cefalexin (250 mg, 2 spoons for every 8 hours) for 1 week postoperatively. Patients were visited 1 week, 2 weeks, 1 month,3 months, 6 months and 12 months postoperatively. MMF was released 2 weeks postoperatively. Heavy gauge elastics were applied for patients without a tight occlusion after release of MMF for 1 week. Malocclusion, infection, sensory disturbances associated with surgery, malunion, additional use of MMF was examined in the patients. In our study delayed union was diagnosed by eliciting pain when the fracture site was torqued and additional MMF was applied for patients with this diagnosis for another 2 weeks after release of MMF. The data of both groups were statistically analyzed using the chi-square test.

## 4. Results

We studied 87 patients with favorable mandibular angle fractures in 2 groups (45 patients for the single miniplates group and 42 patients for the double miniplate group). Of the total, 73 patients (83.9%) were males and 14 patients (16.1%) were females. None of our patients had malocclusion or malunion in either group after 12 months. However, lip numbness occurred in 25 patients (55.6%) in the single miniplates group and 20 patients (47.6%) in the double miniplate group postoperatively without any statistically significant difference (chi-square test: P > 0.46). Additional MMF was applied in 22 patients (48.9%) in the single miniplate group and 18 patients (42.9%) in the double miniplate group with no significant difference (chi-square test: P > 0.57). Furthermore, infection occurred in 3 patients (6.7%) in the single miniplate group and 1 patient (2.4%) in the double miniplate group without any statistically significant differences (chi-square test: P > 0.34).Thirty patients (66.7%) were identified to have one or more complications in the single miniplate group while 59.5% (25 patients) of patients treated with 2 miniplates showed at least one postoperative complication. No significant difference was observed between the two groups regarding incidence of overall complications (chi-square test: P > 0.49).Complications were found to be slightly higher in the patients receiving a single miniplate compared with double miniplates although with no significant difference.

## 5. Discussion

The use of the non-compression monocortical miniplate fixation for the osteosynthesis of mandibular fractures was advocated by Michelet and Champy (12, 13). Champy et al. (1978) reported fixation of the angle fractures on the mandibular superior border by means of a non-compression plate to produce a successful outcome (14). Non-compressive miniplate fixation of angle fractures has gained popularity as a standard treatment approach in different health centers due to its low morbidity and complications (14-16). In addition, some in vitro studies suggest that using a second miniplate along the inferior border theoretically creates a second osteosynthesis line and helps stabilize the fixation protecting the fractures against rotation and torsion (16, 17); however, treatment-related complications from using2 miniplates was reported to be high (18). Levy et al. (1991) demonstrated two monocortical miniplates to have lower complications than a single miniplate for internal fixation of mandibular angle fractures (19). On the contrary, Ellis and Walker showed that using a single miniplate is associated with a lower complication rate than double miniplates in the fixation of angle fractures (8, 20). Postoperative complications were found to be similarly occurring in patients treated with one and two miniplates for favorable mandibular angle fractures. Patients treated with a single miniplate at the superior border were noted to develop lip numbness in 55.6%. Furthermore, 47.6% of patients demonstrated the aforementioned complication when treated with two similar miniplates at the superior border and lateral aspect of the angle,. Fox et al. (2003) studied complications in patients treated with 2-miniplate fixation for mandibular angle fractures and reported the incidence of damage to inferior alveolar nerve in 4.4% of their patients (12). Siddiqui et al. (2007) prospectively studied the complications of single and double miniplate fixation for the treatment of mandibular angle fractures and found subjective lip numbness in 42% of the single miniplate group and 39% of the double miniplate group (16).The sensory nerve disturbances identified after surgery are possibly due to the manipulations at the fracture site during the surgery. The nerve damages were also likely to occur subsequent to the movements of the fractured segments, most of them being transient, as Ellis et al. (2000) showed 17.2% of total facial nerve disturbances improved after 6 weeks and complete healing after 6 months (21).In our study 48.9% of single miniplate patients and 42.9% of double miniplate patients required additional use of MMF. In the study of Siddiqui et al. (2007) additional use of MMF was required in 8% of the single miniplate group and 4% in the double miniplate group (16). In our study infection occurred in 3 patients (6.7%) in the single miniplate group and 1 patient (2.4%) in the double miniplate group. In the study of Danda (2010) infection was noted in 3.7% of single miniplate and 7.4% of double miniplated patients (22). Siddiqui et al. (2007) showed double miniplate patients developed a higher incidence of infection than single miniplates (15% vs. 11%) (16).Levy et al. (1991) reported only a 3.1% complication rate (infection) when using 2 miniplates for the treatment of mandibular angle fractures compared to 26.3% using 1 miniplate (19). The infection rate reported in our study is similar to that of Levy et al. (1991). Ellis and Walker (1994) showed 25% infection rate when using double miniplates for treating mandibular angle fractures (8) arguing that the third molar extractions in the fracture line was the main reason for the higher complication rate, although other factors were also important. One of our study limitations is that we did not determine the effect of third molar removal and placement of drains on infection rate. None of our patients showed signs of malunion. Malunion is associated with decreased blood supply to the area, following mandibular fracture treatment (23). Siddiqui et al. (2007) showed no cases of malunion (16), however, Passeri et al. (1993) reported 1%-2% malunion when studying 82 patients (24). Danda (2010) studied the outcomes of single and double miniplate fixation for the treatment of angle fractures and showed no signs of malocclusion in either group (23) which is similar to the results of our study regarding occlusal findings. No significant difference was observed between the groups regarding overall complication rate. Ellis and Walker (1994) showed 28% overall complication rate when using double miniplates for treating mandibular angle fractures (8). These authors reported a 16% incidence of complications when using a single miniplate in another study (20) which was lower than the results of our study. The technique for placing a single miniplate at the upper border to fix fractures of the mandibular angle was based on the tension lines of the fracture as proposed by Michelet and Champy et al. (16). In vitro studies have reported that in the absence of a second miniplate and under functional loading, a gap appeared at the lower border. The second miniplate theoretically establishes a second line of osteosynthesis, which protects the fracture site against torsion and bending, and provides increased stability. Whether this gap is important to the clinical outcome remains to be seen (16). Closed reduction and MMF is a commonly accepted method for treatment of favorable mandibular angle fractures and obviously has a place in the treatment options but the major disadvantage of this method is that the patient has to survive on a liquid diet for 4 to 6 weeks and oral cleaning cannot be properly done due to a closed mouth. We have shown that two miniplates are no more effective than one in the treatment of favorable mandibular angle fractures. The cost of two miniplates (the cost of fixation devices) for treating mandibular angle fractures in addition to the extra time for the surgery and hospital expenses, point to the fact that two miniplates are not necessary for treatment of favorable mandibular angle fractures. However, further studies are warranted.
